# Pretreatment with 6-Gingerol Ameliorates Sepsis-Induced Immune Dysfunction by Regulating the Cytokine Balance and Reducing Lymphocyte Apoptosis

**DOI:** 10.1155/2021/5427153

**Published:** 2021-12-29

**Authors:** Seong-A Ju, Quang-Tam Nguyen, Thu-Ha T. Nguyen, Jae-Hee Suh, Won G. An, Zak Callaway, Yeonsoo Joe, Hun Taeg Chung, Byung-Sam Kim

**Affiliations:** ^1^School of Biological Sciences, University of Ulsan, Ulsan 44610, Republic of Korea; ^2^Department of Pathology, Ulsan University Hospital, University of Ulsan College of Medicine, Ulsan, Republic of Korea; ^3^Division of Pharmacology, School of Korean Medicine, Pusan National University, Yangsan 50612, Republic of Korea

## Abstract

Sepsis is characterized by an initial net hyperinflammatory response, followed by a period of immunosuppression, termed immunoparalysis. During this immunosuppressive phase, patients may have difficulty eradicating invading pathogens and are susceptible to life-threatening secondary hospital-acquired infections. Due to progress in antimicrobial treatment and supportive care, most patients survive early sepsis. Mortality is more frequently attributed to subsequent secondary nosocomial infections and multiorgan system failure. 6-Gingerol is the major pharmacologically active component of ginger. Although it is known to exhibit a variety of biological activities, including anti-inflammation and antioxidation, the role of 6-gingerol in sepsis-induced immune dysfunction remains elusive. Thus, we investigated whether 6-gingerol improves septic host response to infections during sepsis. 6-Gingerol-treated mice showed significantly lower mortality in polymicrobial sepsis induced by cecal ligation and puncture LPS via enhanced bacterial clearance in the peritoneum, blood, and organs (liver, spleen, and kidney) and inhibited the production of TNF-*α* and IL-6 in TLR2 and/or TLR4-stimulated macrophages. In addition, we demonstrated that survival improvement of secondary infection following septic insult was associated with an initial response of enhanced neutrophil numbers and function at the infection site, reduced apoptosis of immune cells, and a shift from a T helper cell type 2 (Th2) to a T helper cell type 1 (Th1) cytokine balance in the hypoinflammation phase. Our overall findings suggest that 6-gingerol potentially restores sepsis-induced immune dysfunction by shifting the balance of Th1/Th2 and by regulating apoptosis of immune cells.

## 1. Introduction

The immune response in sepsis can be characterized by a proinflammatory cytokine-mediated hyperinflammatory phase and subsequent hypoinflammatory phase with significant immunosuppression [1–3]. Patients fail to eradicate invading pathogens and are susceptible to opportunistic organisms, particularly bacterial infection of the lungs in the hypoinflammatory phase [4, 5]. Over 70% of sepsis-related deaths occur during this immunosuppressive phase, typically due to secondary (opportunistic) infections such as bacterial pneumonia [4–6]. Increasing incidence of nosocomial infections and the emergence of multidrug-resistant bacteria require the study of immunomodulators in the prevention and treatment of sepsis.

Despite the development of powerful antibiotics and specialized treatment available in intensive care units, sepsis remains a serious disorder with high rates of morbidity and mortality. Sepsis, septic shock, and the ensuing multiple organ failure continue to be the most common causes of mortality in surgical intensive care units [7]. Thus, several laboratories have sought an increased understanding of sepsis and septic shock to develop new therapeutic agents that can either reduce or prevent this ever-increasing problem in surgical critical care. The cecal ligation and puncture (CLP) rodent model has been developed as a suitable animal model for polymicrobial sepsis in humans since it can produce symptoms (i.e., appendicitis and peritonitis) similar to those of patients with sepsis [7]. The CLP model produces a hyperdynamic, hypermetabolic state that can lead to a hypodynamic, hypometabolic stage and eventual death. The early hyperdynamic stage is characterized by enhanced cardiac output, tissue perfusion, and reduced vascular resistance. The hallmark of this early stage is the proinflammatory state mediated primarily by neutrophils and macrophages, stimulated by bacteria and/or their toxins. The late hypodynamic stage includes reduced tissue and microvascular blood flow, decreased cardiac function, and enhanced indices of organ injury and dysfunction. The CLP model has been used extensively to investigate clinical manifestations of sepsis and septic shock [8, 9].

6-Gingerol (1-[4′-hydroxy-3′-methoxyphenyl]-5-hydroxy-3-decanone) is the major pungent compound in ginger (*Zingiber officinale*), which has many reported pharmacological effects, including inflammation, indigestion, and hypertension [9–11]. Several studies have demonstrated that 6-gingerol treatment reduced the production of proinflammatory cytokines such as TNF-*α*, IL-6, and IL-1 from LPS-stimulated macrophages [12–14]. In this study, we investigated the effect of 6-gingerol in a CLP-induced polymicrobial sepsis model, which involves various Gram-negative and Gram-positive bacteria. We found that 6-gingerol prevented polymicrobial sepsis induced by CLP through increased bacterial clearance and inhibited inflammatory cytokine production. In addition, we demonstrated that 6-gingerol improved survival of secondary infection following septic insult using the second-hit pneumonia model of sepsis. The well-accepted and widely used CLP model was used as the first hit, and *Pseudomonas aeruginosa* was used to induce pneumonia in mice after CLP as a second hit [15]. CLP induces an immunosuppressive state that leaves the host susceptible to a secondary infection [15–17]. *P. aeruginosa* has been shown to cause little to no mortality when administered alone but was shown to induce a high level of mortality when delivered as a secondary injury in a mouse model of primary peritonitis [15–17]. Therefore, we investigated the protective effect of 6-gingerol from infection, as well as its immune regulation for Th1/Th2 cytokine balance in sepsis, which helps to provide scientific insight for the development of this compound as a drug for the regulation of immune cells in sepsis. Our results demonstrate the value of developing this compound as a drug for regulating immune cells in sepsis.

## 2. Materials and Methods

### 2.1. Reagents

6-Gingerol (Sigma-Aldrich, St Louis, MO) was dissolved to 3.5 *μ*g/ml of 50% ethanol and diluted with drinking water, as required (the final ethanol concentration was less than 0.01%). The following antibodies were purchased from BD Pharmingen, San Diego, CA: phycoerythrin- (PE-) conjugated anti-Ly6G, fluorescein isothiocyanate- (FITC-) conjugated anti-CD11b, PE-conjugated anti-CXCR2, FITC-conjugated anti-CD4, FITC-conjugated anti-CD8, PE-conjugated anti-IFN-*γ*, PE-conjugated anti-CD4, PE-conjugated anti-CD8, and FITC-conjugated antiannexin V antibodies. *P. aeruginosa* (ATCC27853) was obtained from the American Type Culture Collection (ATCC). For the M1 and M2 macrophage analysis, cells were separately stained with monoclonal antibodies specific for F4/80-PE from eBioscience; F4/80-FITC and CD206-PerCP/Cy5.5 from BioLegend, San Diego, CA; and CD80-FITC and iNOS-FITC from BD Transduction Laboratories, San Diego, CA. Bacteria were grown in brain heart infusion (BHI) media (Difco) at 37°C for 18 h, and aliquots were frozen at −80°C.

### 2.2. Model of Polymicrobial Sepsis

BALB/c (7~8 weeks of age, males, average weight: 20 g) mice (Orient Bio-Charles River, Seoul, Republic of Korea) were maintained under specific pathogen-free conditions at 22°C. All animal experiments were conducted according to the regulations of the Animal Committee of the University of Ulsan. For cecal ligation and puncture (CLP), mice were anesthetized via intraperitoneal injection of a mixture of Zoletil 50 (30 mg/kg; Virbac Carros, France) and Rompun (10 mg/kg; Bayer Korea Ltd., Seoul, Korea). Under sterile conditions, a 1-2 cm incision was made in the lower left abdomen, and the cecum was exposed. The distal portion of the cecum was ligated with a 4-0 silk suture, punctured once with a 26- or 21-gauge (G) needle to induce moderate or severe sepsis, respectively [18]. For the second-hit pneumonia model of sepsis, to create a long-term infection with a relatively low mortality (<10%), we used cecal ligation and punctured once with a 29-gauge (G) needle [19]. The abdomen was closed in two layers, and 1 ml of 0.9% saline was administered subcutaneously (s.c.). Sham-operated mice were subjected to similar laparotomy without CLP. For the LPS or *Escherichia coli* (*E. coli*) model, LPS (150 *μ*g/mouse) or *E. coli* (2 × 10^7^ cells/mouse) was injected i.p., respectively. Survival was recorded every 12 h for 7 days. Mice were injected intraperitoneally with 6-gingerol (50 *μ*g/mouse) or control (0.01% ethanol/mouse) at 2 days before CLP.

Mice were fed drinking water containing 3.5 *μ*g/ml of 6-gingerol every day for 5 days before CLP, while control mice were fed drinking water containing 0.01% ethanol until the end of the experiment. Freshly made 6-gingerol-containing drinking water was provided every 2 days.

### 2.3. Analysis of Blood and Cells in the Peritoneal Cavity

At 4 h and 24 h after surgery, two punctures with a 26-gauge needle were made for CLP; then, mice (*n* ≥ 5 mice/group) were killed and blood was collected and stored for analysis of bacteria and cytokines. The peritoneal cavities were washed with 3 ml of sterile phosphate-buffered saline (PBS), and the lavage fluids were collected. After a 10 *μ*l aliquot of the lavage fluid was removed for assessment of bacterial colony-forming units (CFUs), the remaining fluid was centrifuged at 400 × *g* for 5 min at 4°C, and the supernatants were collected and stored at -80°C to determine cytokine concentrations. Cell pellets were treated with red blood cell lysing buffer, washed twice, and resuspended in PBS containing 3 mM EDTA. For flow cytometry, cells were incubated with 1 *μ*g/ml of antimouse Fc*γ* MAb (clone 2.4G2) for 15 min to block nonspecific binding of mAbs and then stained with 1 *μ*g/ml of PE-conjugated anti-Ly6G, F4/80, B220, or FITC-conjugated anti-CD11b, CD3, and annexin V antibodies and analyzed with FACS. CD11b^+^ Ly6G^+^ cells were considered as neutrophils and CD11b^+^ F4/80^+^ cells as macrophages.

### 2.4. Induction of Secondary Bacterial Pneumonia

To create a relevant model of secondary bacterial pneumonia, on Day 4 post-CLP, the surviving mice were anesthetized, as mentioned above. *P. aeruginosa* (ATCC 27853) suspension was used as the secondary injury [19]. Using a pipette, 20 *μ*l of a *P. aeruginosa* suspension (8 × 10^7^ CFU/mouse) was slowly injected intranasally (divided equally between the nares), observed to be aspirated on inhalation, and held in position for 1 min. Mice were fed drinking water containing 3.5 *μ*g/ml of 6-gingerol, while control mice were fed drinking water containing 0.01% ethanol from Day 5 pre-CLP until the end of the secondary bacterial pneumonia experiment. Freshly made 6-gingerol-containing drinking water was provided every 2 days. Survival was recorded for 14 days following pneumonia. Animals were then sacrificed 4 days post-CLP or 4 h and 24 h after secondary injury.

### 2.5. Bronchoalveolar Lavage

Mice were anesthetized as mentioned previously. For bronchoalveolar lavage (BAL), animals were placed supine and the trachea was exposed by dissection. A 24-gauge angiocatheter was inserted into the trachea, and 1 ml of sterile saline was injected and immediately withdrawn. BAL fluid was placed in sterile vials and analyzed for cytokine levels, bacteria count, and immune cells.

### 2.6. CFU Assay

Lungs were collected in sterile phosphate-buffered saline (PBS), and extracted lobes were homogenized and serially diluted. BAL fluid or lung tissue homogenate was spread onto *BHI agar* (Difco) plates. Following overnight culture in a 37°C incubator, colonies were counted and the bacteria number was calculated. Bacteria numbers in BAL and lung were indicated as CFU/ml BAL and CFU/ml lung tissue, respectively.

### 2.7. Isolation of Cells and Culture

Peritoneal macrophages were isolated as previously described [20]. Peritoneal exudated cells were harvested by lavaging the peritoneal cavity with 10 ml sterile ice-cold PBS. Contaminating erythrocytes were lysed by washing cells in red blood cell lysing buffer. Cells were washed three times and resuspended in RPMI-1640 medium supplemented with 10% heat-inactivated fetal bovine serum (FBS). Cells were then cultured in plastic tissue culture flasks at 37°C in a CO_2_ incubator for 2 hours. The cultures were then washed three times to remove all nonadherent cells; 95% of the adherent cell population was macrophages as determined by flow cytometry. Mouse neutrophils were isolated from the bone marrow by MACS using the Anti-Ly6G MicroBead Kit (Miltenyi Biotec, Auburn, CA) according to the manufacturer's protocols. Isolated neutrophils were 98% pure, as assessed by flow cytometry.

Macrophages and neutrophils (5 × 10^5^ cells/well) in RPMI-1640 medium containing 10% FBS were cultured with control, 6-gingerol (1 *μ*g/ml), Pam3 (500 ng/ml), or LPS (500 ng/ml).

### 2.8. Cell Depletion In Vivo

For macrophage depletion, mice were injected i.p. with clodronate liposomes (Clodrolip) (2 mg/20 g) on day 1.5 before polymicrobial sepsis induced by CLP (Day 0). Empty liposomes were used as the control. Clodrolip contains approximately 18 ± 2 mg of clodronate per milliliter (0.5 micromoles of clodronic acid disodium salt tetrahydrate, CH_2_Cl_2_Na_2_O_6_P_2_·4H_2_O). After Clodrolip injection, liposomes were phagocytosed and digested by macrophages, followed by intracellular release and accumulation of clodronate, causing >90% depletion of macrophages within 24–36 h [21].

### 2.9. Cytokine Analysis

Cytokines in BAL were quantified using a cytometric bead array (CBA) kit mouse inflammation (BD Biosciences, San Diego, CA), according to the manufacturer's protocol. It was analyzed on a FACSCanto™ II (BD Biosciences, San Diego, CA) with FACSDiva software. IL-17 production was assessed according to the manufacturer's protocol (mouse IL-17 DuoSet ELISA, R&D Systems).

### 2.10. Assessment of Apoptosis

Apoptosis was evaluated by a FACScan flow cytometer (BD Biosciences, San Diego, CA) using the Annexin V-FITC kit (BD Biosciences).

### 2.11. Determination of Intracellular Cytokines

Splenocytes were cultured for 6 h with phorbol myristate acetate (PMA, 50 ng/ml) and ionomycin (500 ng/ml), and cytokine release was prevented by treatment with Golgi-stop (BD Pharmingen, San Diego, CA). Following surface staining for CD4^+^ or CD8^+^, cells were fixed in Cytofix/Cytoperm solution (BD Pharmingen) and stained with PE-conjugated antimouse IFN-*γ* (BD Pharmingen). Finally, cells were analyzed on a FACSCanto™ II (BD Biosciences) with FACSDiva software.

### 2.12. Bacterial Killing Assays

The killing of bacteria was measured as described previously [22]. Briefly, macrophages were mixed with opsonized *E. coli* at an MOI of 40 : 1 and incubated at 37°C for 10 min with continuous rotation. Noningested bacteria were discarded by centrifugation, and cells were cultured with 6-gingerol for the next 60 min with slow rotation. Killing was stopped by spinning the cells onto ice after the addition of 1 ml distilled water containing 0.01% bovine serum albumin, and the number of viable bacteria was determined by plating tenfold serial dilutions. The percent killing was calculated as [1 − (number of viable bacteria at 60 min/number of viable bacteria at 0 min)] × 100.

### 2.13. Measurements of ROS Generation

Purified macrophages were stained with 2 *μ*M 2′,7′-dichlorodihydrofluorescein diacetate (Molecular Probes, MO) for 20 min at 37°C in the dark. After incubation, the macrophages were washed twice with PBS, incubated with control or 6-gingerol (1 *μ*g/ml) and *E. coli* in an MOI of 20 : 1 at 37°C for 1 h, and analyzed by FACS. Reactive oxygen species (ROS) production was expressed as mean fluorescence intensity (MFI). To prevent ROS production, macrophages were preincubated with 10 *μ*M of nicotinamide adenine dinucleotide phosphate reduced (NADPH) oxidase inhibitor diphenyleneiodonium chloride (DPI; Sigma) for 1 h at 37°C before the addition of 6-gingerol.

### 2.14. Histology

For histological examination, lung tissue was fixed with 10% neutral buffered formaldehyde and embedded in paraffin. The section was deparaffinized and stained with hematoxylin and eosin (H&E) solution.

### 2.15. Statistical Analysis

All data were analyzed using GraphPad Prism 5 (GraphPad Software, San Diego, CA, USA). Data obtained from two and three groups were analyzed by *t*-test and one-way ANOVA with post hoc Tukey's test, respectively. Survival curves were compared by using the log-rank test.

## 3. Results

### 3.1. Administration of 6-Gingerol Protected against Sepsis-Induced Mortality

**Figure 1:**
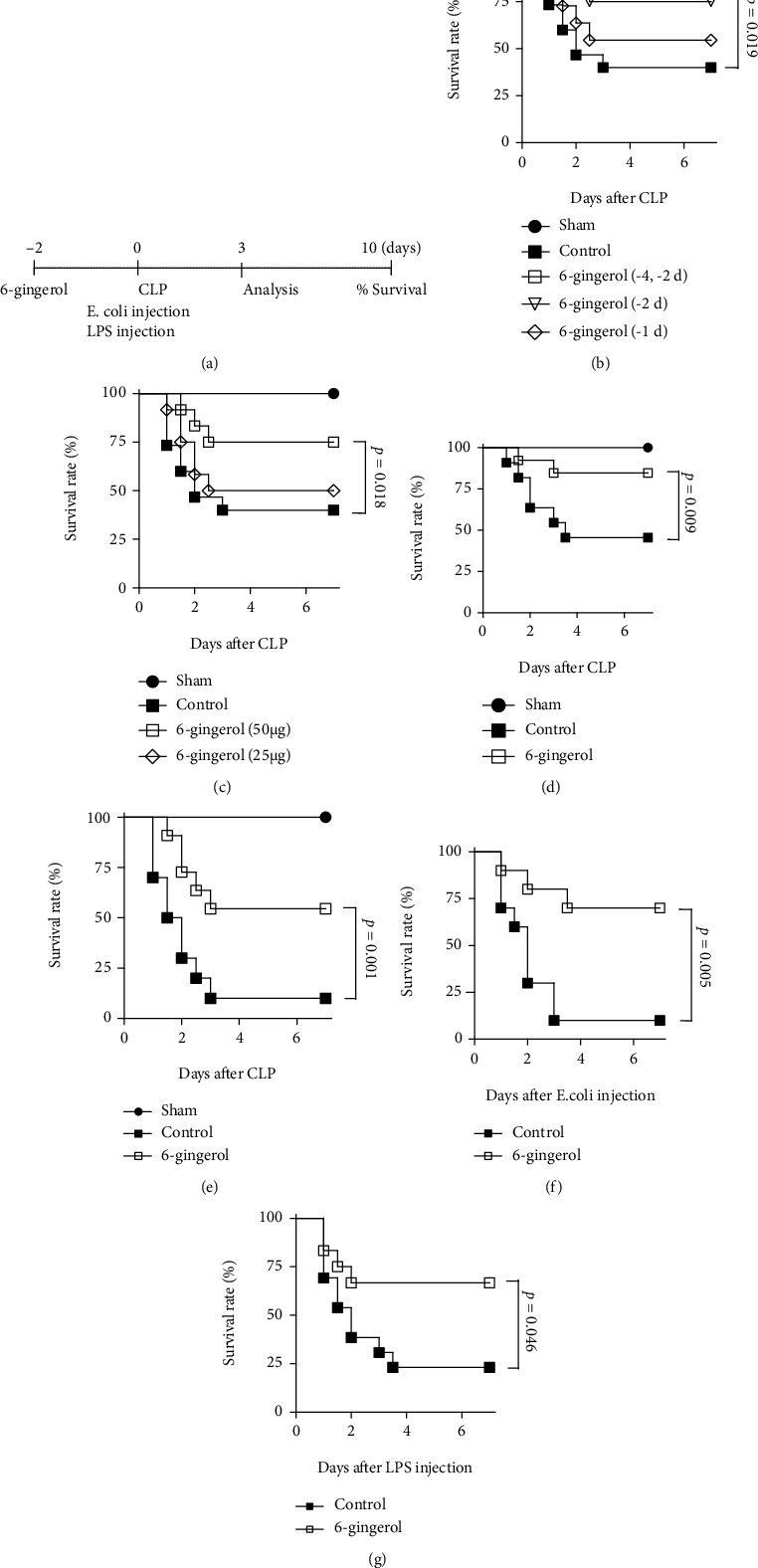
Administration of 6-gingerol protected mice from septic death. (a) Schematic diagram for the model of polymicrobial sepsis. (b–d) Moderate sepsis in mice was induced using 26-gauge needles with two punctures on Day 0. (b) 6-Gingerol (50 *μ*g) was treated at indicated times before CLP. (c) 6-Gingerol was i.p. injected into mice at indicated dose before CLP. (d) Mice were fed drinking water containing 3.5 *μ*g/ml of 6-gingerol, while control mice were fed drinking water containing 0.01% ethanol every day for 7 days before CLP. (e) Mice were i.p. injected with control or 6-gingerol (50 *μ*g). After 2 days, mice were subjected to severe CLP using 21-gauge needles with two punctures. (f, g) Sham mice were subjected to only laparotomy without CLP. Control or 6-gingerol (50 *μ*g) treatment was i.p. injected into mice Day 2 before peritoneal injection with *E. coli* (2 × 10^7^ cells/mouse) (f) or with LPS (150 *μ*g/mouse) (g). Mouse survival was monitored every 12 h for 7 days. Each group contained 10 to 15 mice, and results of two or three experiments were pooled. The curve comparison with the log-rank (Mantel-Cox) test revealed statistically significant differences as shown on the graph.

To investigate whether 6-gingerol has a protective effect on polymicrobial sepsis, CLP was performed on BALB/c mice, and their survival was monitored for up to 7 days. In the moderate CLP experiment, mice were subjected to CLP using 26-gauge needles and two punctures. To examine the effect of 6-gingerol treatment, mice were i.p. injected with 6-gingerol (50 *μ*g/mouse) or control (0.01% ethanol) before CLP. As shown in Figure 1(b), treatment with 6-gingerol 2 days before CLP significantly increased mouse survival, compared with the control. By Day 7 post-CLP, 75% of the 6-gingerol-treated mice were still alive (9 of 12 mice), compared to 40% of the control-treated mice (6 of 15 mice). When 6-gingerol was injected 2 and 4 days before CLP, the protective effect was similar to that observed following injection of 6-gingerol 2 days before CLP.  Figure 1(c) shows that treatment with 6-gingerol significantly increased mice survival in a dose-dependent manner. On Day 7 post-CLP, the survival rate for mice treated with 50 *μ*g/mouse 6-gingerol was 75% (9 of 12 mice), while that for mice treated with 25 *μ*g/mouse 6-gingerol was 50% (6 of 12 mice). For mice fed drinking water containing 3.5 *μ*g/ml of 6-gingerol every day for 10 days before CLP, survival also increased, compared with the control (Figure 1(d)). Only 45% of the control-treated mice (5 of 11 mice) survived, compared to 85% of the 6-gingerol-treated mice (11 of 13 mice). In the severe sepsis experiment (using 21 G needles and two punctures), the 6-gingerol-treated mice were also more resistant to sepsis than control-treated mice (Figure 1(e)). On Day 7 post-CLP, 55% of the 6-gingerol-treated mice (6 of 11 mice) were still alive, compared to 10% of the control-treated mice (1 of 10 mice).

Sham surgery did not cause any mortality, and the survival of 6-gingerol-treated mice with a C57BL/6 background with CLP-induced sepsis was also higher than that of control-treated C57BL/6 mice with CLP-induced sepsis, indicating the 6-gingerol effects were not mouse strain-specific (data not shown). *E. coli* is the most common cause of Gram-negative bacteria-induced sepsis. Similarly, LPS-induced endotoxemia is marked by the activation of inflammatory responses, which can lead to shock, multiple organ damage, and even death. Therefore, *E. coli* and LPS were used to evaluate the protective effects of 6-gingerol in other sepsis mouse models.  Figure 1(f) showed that treatment with 6-gingerol increased the survival of *E. coli*-infected mice. On Day 7 post-*E. coli* infection, the survival rate for mice treated with 6-gingerol was 70% (7 of 10 mice), while that for control mice was only 10% (1 of 10 mice). Moreover, 6-gingerol treatment also reduced the mortality of LPS-injected mice (Figure 1(g)). The survival rate for 6-gingerol-treated mice on Day 7 was 66% (8 of 12 mice) in septic shock induced by LPS, while survival in control-treated mice was only 23% (3 of 13 mice).

### 3.2. Administration of 6-Gingerol Increased Immune Cell Infiltration and Bacteria Clearance and Reduced Cell Apoptosis

**Figure 2:**
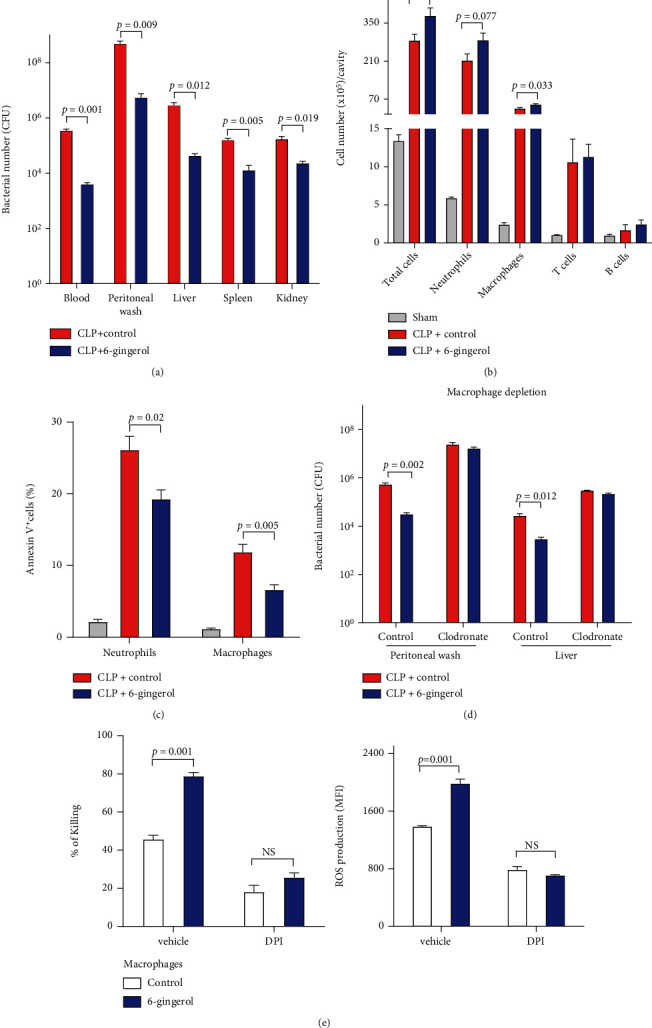
Administration of 6-gingerol increased immune cell infiltration and bacteria clearance and reduced cell apoptosis. Mice were i.p. injected with control or 6-gingerol (50 *μ*g). After 2 days, mice were subjected to CLP using 26-gauge needles with two punctures. (a–c) Bacterial load (a), cell recruitment to peritoneum (b), and cell apoptosis (c) were determined at 24 h after CLP, as described in Materials and Methods. (d) Mice were i.p. injected with control or 6-gingerol (50 *μ*g) on Day 2 before CLP and then injected with clodronate liposomes to deplete macrophages. After depletion, mice were subjected to CLP using 26-gauge needles with single punctures on Day 0. Twenty hours after CLP, bacterial load was evaluated by colony plating assay. (e) Macrophages were preincubated with ROS inhibitor DPI (10 *μ*M) for 1 h before 6-gingerol (1 *μ*g/ml) treatment. Bacterial killing activities (left panel) and ROS production (right panel) by macrophages against *E. coli* were determined. Data are the means and standard errors of means obtained for individual mice. Data are representative of at least three independent experiments in which each group contained four to six mice. Statistically significant differences are shown on the graph. NS: not significant.

We investigated whether the survival of 6-gingerol-treated mice was higher than that of the control-treated mice, and whether survival was associated with enhanced bacterial clearance. As shown in Figure 2(a), bacterial clearance in the organs of 6-gingerol-treated mice in the CLP model was greater than that in the organs of control-treated mice. At 24 h post-CLP, CFUs of bacteria in the blood, peritoneum, and organs (liver, spleen, and kidney) of 6-gingerol-treated mice were 66 to 88 times lower than those in the control-treated mice.

In the CLP-induced sepsis model, neutrophils and macrophages are the first recruited into the peritoneum and play an important role in host defense [23–25]. As shown in Figure 2(b), at 24 h post-CLP, 6-gingerol-treated mice had a higher number of infiltrated peritoneal cells, including neutrophils and macrophages, than in control-treated mice. T- and B cell numbers were similar between 6-gingerol-treated mice and control-treated mice.

Apoptosis of neutrophils and macrophages in sepsis induces defects in immunity and has been considered a critical factor in determining sepsis-induced mortality. As shown in Figure 2(c), CLP-induced sepsis caused neutrophil and macrophage apoptosis in the peritoneum; however, this effect was inhibited by 6-gingerol treatment. The 6-gingerol-treated mice had a lower level of annexin V expression on neutrophils and macrophages than in control-treated mice. To determine whether macrophages were required for resistance to CLP-induced sepsis in 6-gingerol-treated mice, we depleted macrophages by *in vivo* injection of clodronate liposomes. Following macrophage depletion, the bacterial load in the peritoneum or liver did not change in comparison to the control-treated mice (Figure 2(d)). In addition, macrophages ingest microorganisms via phagocytosis, and the ingested bacteria were killed by ROS derived from superoxide, which is produced by an activated, phagosome-bound NADH oxidase [25–27]. We evaluated whether the enhanced bacterial clearance in 6-gingerol-treated macrophages was involved in ROS generation. Cells were preincubated with ROS inhibitor, DPI before incubation with 6-gingerol, and *E. coli*, and antibacterial activities were determined. As shown in Figure 2(e), 6-gingerol enhanced bacterial killing activity and ROS production by up to 68% or 65% in macrophages, respectively. However, DPI treatment inhibited 6-gingerol-induced effects. This indicates that 6-gingerol-induced antibacterial activities are, in part, regulated by the production of ROS in macrophages. Our results thus support the notion that macrophages may play a critical role in the resistance to CLP-induced sepsis in 6-gingerol-treated mice.

### 3.3. 6-Gingerol Inhibited Production of Inflammatory Cytokines

**Figure 3:**
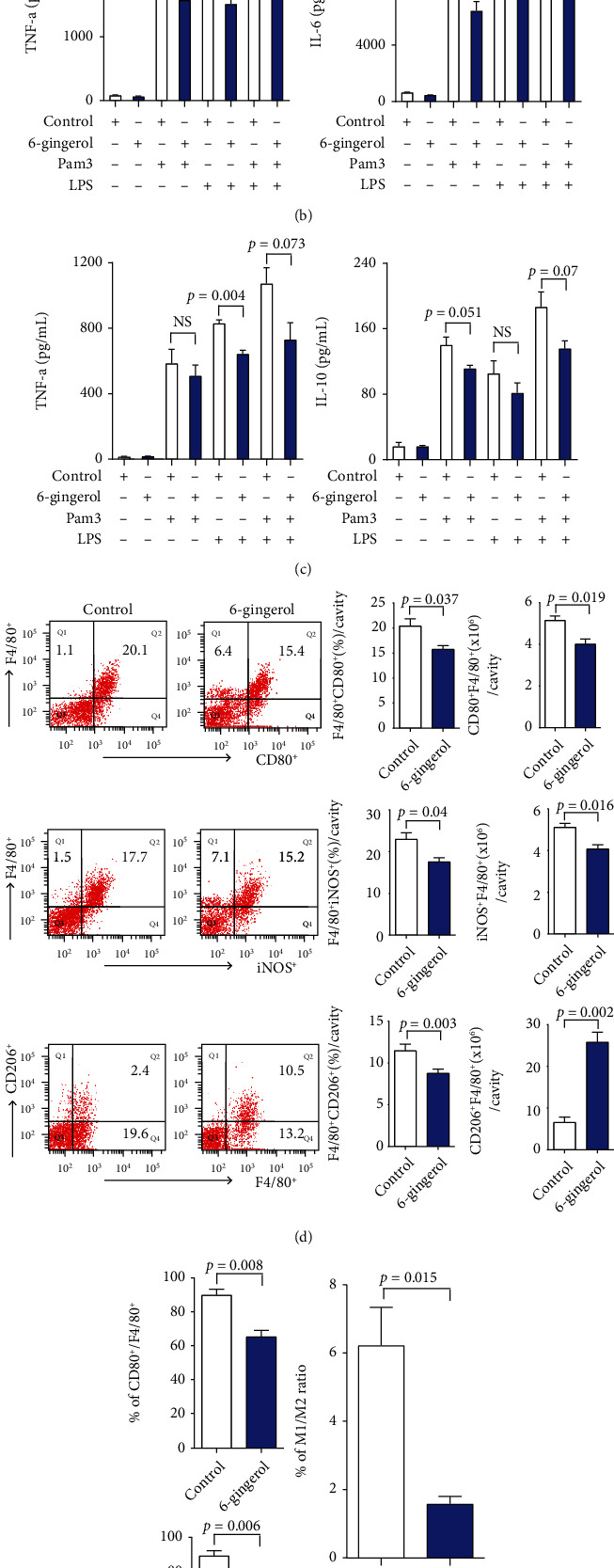
6-Gingerol inhibited the production of inflammatory cytokines. (a) Mice were i.p. injected with control or 6-gingerol (50 *μ*g). After 2 days, mice were subjected to CLP using 26-gauge needles with two punctures. Blood was collected at 4 or 24 h after CLP, and their peritonea were washed with 3 ml of PBS. After centrifugation at 400 × g, 5 min, cytokine concentrations in the peritoneum (top panels) and serum (bottom panels) were determined using a CBA kit; ND: not detected. Purified macrophages (b) and neutrophils (c) were cultured with combinations of control, 6-gingerol (1 *μ*g/ml), Pam3 (500 ng/ml), and LPS (500 ng/ml), as indicated. After 24 h, supernatants were harvested, and cytokine levels were measured with the CBA kit. Data are presented as the means of triplicate cultures. (d, e) Mice were i.p. injected with control or 6-gingerol (50 *μ*g). After 2 days, mice were subjected to CLP using 26-gauge needles with two punctures. Peritoneal cells were harvested and analyzed. (d) Representative flow plots (left panels), percentages (middle panels), and numbers (right panels) presented M1 (CD80^+^ F4/80^+^, iNOS^+^ F4/80^+^) or M2 (CD206^+^ F4/80^+^) macrophages in peritoneal cavity and (e) percentages of CD80^+^, iNOS^+^, or CD206^+^ cells in macrophages (F4/80^+^) (left panels). The M1/M2 ratio was calculated based on the percentages (top panel) and cell numbers (bottom panel) of iNOS^+^ F4/80^+^ cells and CD206^+^ F4/80^+^ cells in peritoneal cavity cells, respectively. Error bars indicated the mean ± SD for three separate experiments, *n* = 3 for each group. Statistically significant differences are shown on the graph. NS: not significant.

Sepsis is associated with high cytokine levels and sustained infection, which contribute to multisystem organ failure, the direct cause of mortality [28, 29]. Studies have shown that the production of inflammatory cytokines and chemokines are augmented during CLP-induced sepsis and are associated with mortality when they are expressed at high levels [30, 31]. Therefore, we determined the effect of 6-gingerol treatment on inflammatory cytokine/chemokine levels after CLP. Cytokine profiles of sham, control, and 6-gingerol-treated mice were measured at 4 or 24 h after surgery (Figure 3(a)). Sham surgery did not cause a significant rise in cytokine production. Inflammatory cytokine/chemokine (TNF-*α*, IL-6, MCP-1, and IL-10) levels at 24 h were higher than those at 4 h post-CLP. Inflammatory cytokine/chemokine levels in the peritoneum (Figure 3(a), top panels) and serum (Figure 3(a), bottom panels) of 6-gingerol-treated mice were significantly lower than the levels in the peritoneum and serum of control-treated mice at 24 h post-CLP. Only IL-6 levels in septic 6-gingerol-treated mice were significantly lower than those in control-treated mice at 4 h post-CLP. These results indicate that the inflammatory response to sepsis in 6-gingerol-treated mice was less vigorous than that in control-treated mice, which may be one of the reasons for less severe organ failure and decline in mortality in septic 6-gingerol-treated mice.

Polymicrobial sepsis was induced by CLP, which involves various Gram-negative (G-) and Gram-positive (G+) bacteria. Of all identified TLRs, it is known that TLR2 and TLR4 differentially recognize G+ and G- bacteria. TLR4 recognizes LPS, the most potent immunostimulant of Gram-negative bacteria; whereas, TLR2 plays a major role in detecting G+ bacteria by recognizing lipoproteins and lipoteichoic acid [24]. In order to evaluate the effect of 6-gingerol on inflammatory cytokine production by macrophages and neutrophils, purified cells were incubated with combinations of 6-gingerol, Pam3, and LPS. Cytokines produced in culture supernatants were then determined. The release of TNF-*α* and IL-6 from macrophages (Figure 3(b)) or the release of TNF-*α* and IL-10 from neutrophils (Figure 3(c)) by Pam3 and LPS was inhibited by 6-gingerol treatment. Based on changes to the cytokine profile after 6-gingerol treatment, we investigated the effects of 6-gingerol on M1 and M2 macrophage polarization. Flow cytometry data indicated that 6-gingerol could decrease the ratio of CD80^+^/F4/80^+^ or iNOS^+^/F4/80^+^ and significantly increase the expression of CD206^+^/F4/80^+^ than in the control group (Figures 3(d) and 3(e)). The results suggest that 6-gingerol inhibits sepsis-induced hyperpolarization of M1 and promotes M2 phenotypic alteration in macrophages.

### 3.4. Administration of 6-Gingerol Improved Survival and Bacterial Clearance to Intranasal P. aeruginosa Infection in Postseptic Mice

**Figure 4:**
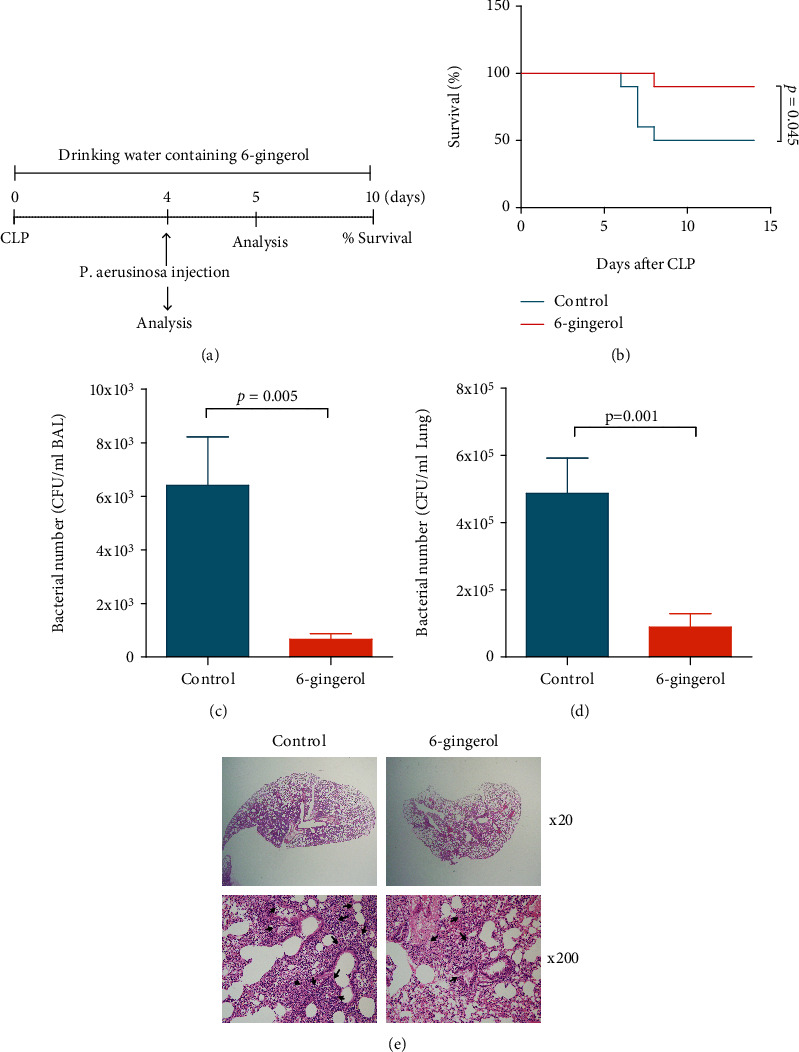
Administration of 6-gingerol improved survival and bacterial clearance to intranasal *P. aeruginosa* infection in postseptic mice. (a) Schematic diagram for induction of secondary bacterial pneumonia. (b) Mice were subjected to CLP using 29-gauge needles and punctured once. From 5 days before CLP to the end of the experiment, mice were fed drinking water containing 3.5 *μ*g/ml of 6-gingerol every day, while control mice were fed drinking water containing 0.01% ethanol. On Day 4 post-CLP, mice were challenged with an intranasal administration of 8 × 10^7^ CFU of *P. aeruginosa*. Mouse survival was monitored every 24 h for 14 days. The curve comparison with the log-rank (Mantel-Cox) test revealed statistically significant differences as shown on the graph (*n* = 10). After CLP, survivors on Day 4 were secondarily challenged by intranasal instillation of *P. aeruginosa* (8 × 10^7^ CFU). (c, d) Bacterial load was determined 24 h after instillation, by quantitative cultures of BAL fluid (c) and lung homogenates (d). (e) Lung damage was assessed by microscopic examinations of hematoxylin and eosin-stained lung sections from control and 6-gingerol-treated mice 24 hours after intranasal inoculation. Data represent the mean ± SEM. Data are representative of at least three independent experiments in which each group contained six mice. Statistically significant differences are shown on the graph.

To investigate the role of 6-gingerol in host defense against secondary *P. aeruginosa* pneumonia after sepsis, we first established that these polymicrobial sepsis-induced mice had survival rates of >90% following CLP. Mice were subjected to CLP, via one puncture with a 29-gauge needle. This level of injury was utilized in order to create a prolonged infection with relatively low mortality (<10%). Mice were administrated 3.5 *μ*g/ml of 6-gingerol contained in drinking water daily. Because each mouse used in our experiments weighed approximately 20 g and consumed 3-4 ml of drinking water per day, dosages of 6-gingerol were 525-700 *μ*g/kg body weight/day. Control mice were fed water containing 0.01% ethanol. Survivors on day 4 after CLP were then subjected to intranasal instillation of a *P. aeruginosa* bacterial load of 1.6 × 10^8^ CFU. Postseptic control mice displayed high susceptibility to secondary pneumonia (mortality rate, 50%). In contrast, 6-gingerol-treated mice displayed resistance to secondary pulmonary infection, with survival rates of 90% (Figure 4(b)).

To understand how 6-gingerol treatment confers protection against secondary *P. aeruginosa* pneumonia, we assessed lung clearance of *P. aeruginosa* through quantitative bacterial cultures of BAL fluids and lung homogenates collected 24 hours after intranasal instillation. Bacterial clearance significantly improved in 6-gingerol-treated mice compared to control mice (Figures 4(c) and 4(d)). In addition, microscopic analysis of lung sections stained with hematoxylin-eosin showed that 6-gingerol treatment attenuated lung damage in secondary *P. aeruginosa* pneumonia. Lung sections of 6-gingerol-treated mice showed significant decreases in extensive foci of consolidation with loss of pulmonary structure (Figure 4(e)). These observations indicate that 6-gingerol enhances the bacterial clearance of intranasal *P. aeruginosa* infection in postseptic mice compared to the control group, which reduced lung tissue damage and was associated with an increased survival rate.

### 3.5. Administration of 6-Gingerol Increased Neutrophil Infiltration into BAL Fluid following Secondary Challenge by Intranasal Instillation of P. aeruginosa

**Figure 5:**
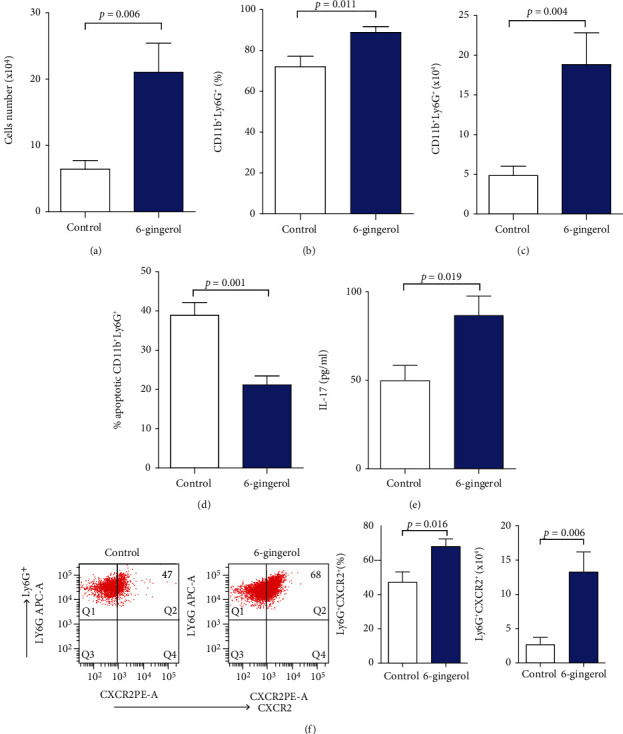
Administration of 6-gingerol increased neutrophil infiltration into BAL fluid and decreased neutrophil apoptosis. Mice were subjected to CLP and fed 6-gingerol as mentioned in the Materials and Methods. Survivors on Day 4 were secondarily challenged by intranasal instillation of *P. aeruginosa* (8 × 10^7^ CFU). (a) White blood cell numbers were assessed in BAL fluid 4 hours after instillation. (b–d) Neutrophil percent (b) and numbers (c), percent of apoptosis, and (d) IL-17 of BAL fluid sup (e) representative flow plots of CXCR2^+^ expression on neutrophils (*left panels*), percentage (*middle panel*), and numbers (*right panel*) (f) in BAL fluid were determined as described in Materials and Methods. Results are representative of three independent experiments and expressed as the mean ± SD. Statistically significant differences are shown on the graph.

To observe the effects of 6-gingerol in host defense against secondary *P. aeruginosa* pneumonia, we collected and analyzed immune cells in BAL fluid 4 hours after secondary *P. aeruginosa* infection. We observed a significant increase in the total number of immune cells and neutrophils in BAL fluid of 6-gingerol-treated mice when compared to control mice. The total number of white blood cells of 6-gingerol-treated mice in BAL fluid was 3.3 times that of control mice (Figure 5(a)). In BAL fluid, most infiltrated cells were neutrophils, and 6-gingerol-treated mice showed an increased percentage and number of neutrophils compared to control mice (Figures 5(b) and 5(c)). Neutrophil numbers in BAL fluid from 6-gingerol-treated mice were 3.9 times greater than those from control mice (Figure 5(c)). Because 6-gingerol-treated mice showed increased neutrophils in BAL fluid compared to control mice, we investigated whether 6-gingerol has antiapoptotic effects *in vivo*. As shown in Figure 5(d), neutrophils harvested from BAL fluid of 6-gingerol-treated mice showed significantly decreased neutrophil apoptosis than those found in control-treated mice. In addition, the migration and microbicidal activity of neutrophils in sepsis is regulated by IL-17 [32, 33]. A decrease in IL-17 is correlated with the risk of bacteremia [34]. Thus, we investigated whether 6-gingerol augments IL-17 levels, resulting in the migration of neutrophils. The 6-gingerol treatment augmented the IL-17 levels in BAL fluid (Figure 5(e)). Besides IL-17, CXCR2 plays a crucial role in the migration of neutrophils to infected sites during sepsis [35–37]. We observed that 6-gingerol increased CXCR2 expression in neutrophils (Figure 5(f)). Therefore, 6-gingerol promotes neutrophil infiltration to the BAL fluid through an increase in IL-17 and CXCR2 levels.

### 3.6. 6-Gingerol Modulated Cytokine Balance toward a Proinflammatory Pattern in Secondary P. aeruginosa-Infected Mice

**Figure 6:**
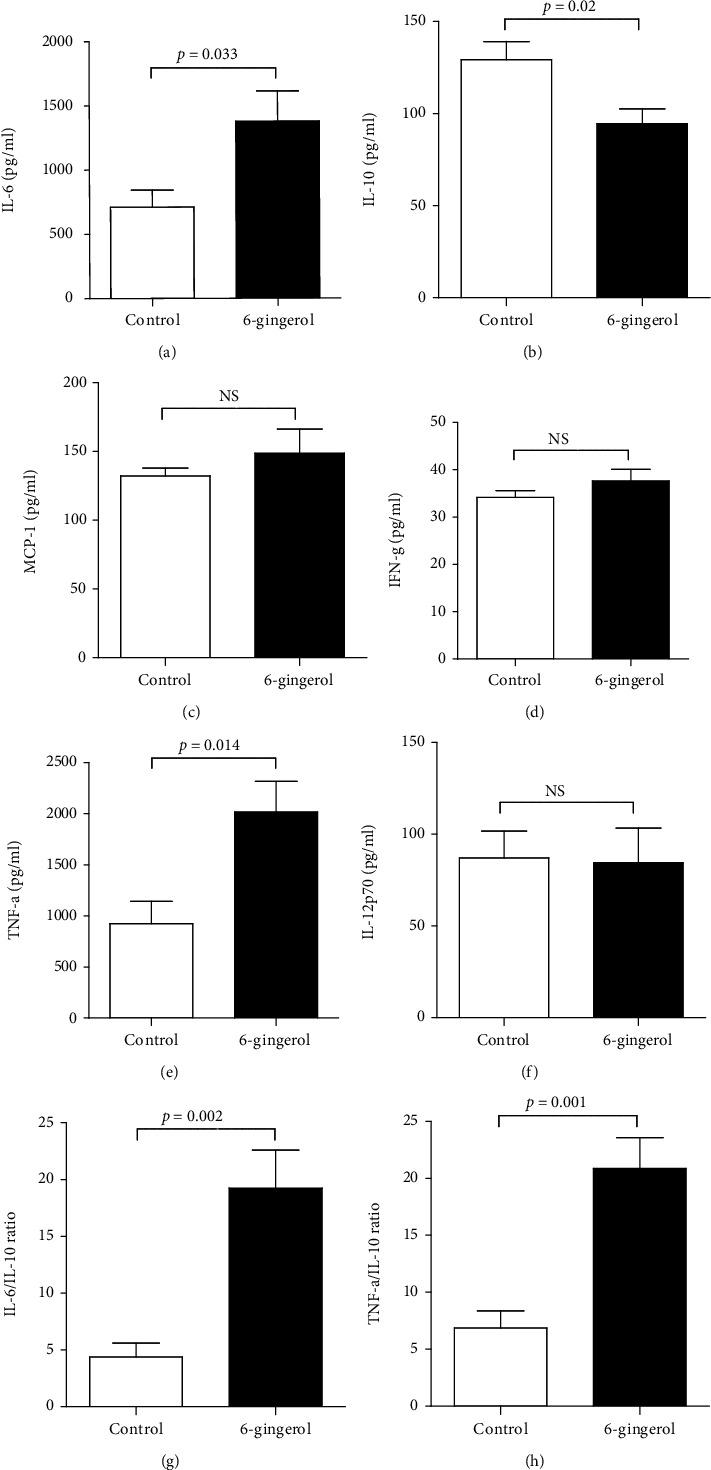
6-Gingerol modulated cytokine balance toward a proinflammatory pattern in secondary *P. aeruginosa-*infected mice. Mice were subjected to CLP and fed 6-gingerol. Survivors were challenged on Day 4 by intranasal instillation of *P. aeruginosa* (8 × 10^7^ CFU). (a–f) Following a second septic insult, evaluation of BAL cytokines, including IL-6 (a), IL-10 (b), MCP-1 (c), IFN-*γ* (d), TNF-*α* (e), and IL-12p70 (f), were measured 24 hours after instillation with the CBA kit. They were analyzed on a FACSCanto™ II (BD Biosciences) with FACSDiva software. (g, h) As an index of overall balance of pro- and anti-inflammatory cytokines, the rate of IL-6 to IL-10 (g) and the rate of TNF-*α* to IL-10 (h) in BAL were calculated. Data are presented as the means of triplicate cultures. Results are representative of three independent experiments and expressed as the mean ± SD. Statistically significant differences are shown on the graph. NS: not significant.

To further investigate the role of 6-gingerol in the immune response to secondary *P. aeruginosa* pneumonia, we assessed pulmonary levels of cytokines involved in antibacterial defense. IL-6 and TNF-*α* levels in BAL fluid from 6-gingerol-treated mice were significantly higher than levels in BAL fluid from controls. In contrast, IL-10 levels in 6-gingerol-treated mice were significantly lower than those in control-treated mice at 24 hours after intranasal instillation (Figures 6(a)–6(f)). As an index of the overall balance of pro- and anti-inflammatory cytokines, ratios of IL-6 to IL-10 and TNF-*α* to IL-10 in BAL fluid were calculated. 6-Gingerol-treated mice showed a shift from a Th2 to a Th1 cytokine balance in the hypoinflammation phase (Figures 6(g) and 6(h)). These results indicate that a secondary *P. aeruginosa* infection in control mice resulted in a shift in the pro- and anti-inflammatory cytokine balance favoring predominantly IL-10 response, while 6-gingerol modulated a cytokine balance toward a proinflammatory pattern.

### 3.7. Splenocytes from 6-Gingerol-Treated Mice Showed Significant Increases in IFN-*γ* Production and Decreased Apoptosis Compared to Control 4 Days Post-CLP

**Figure 7:**
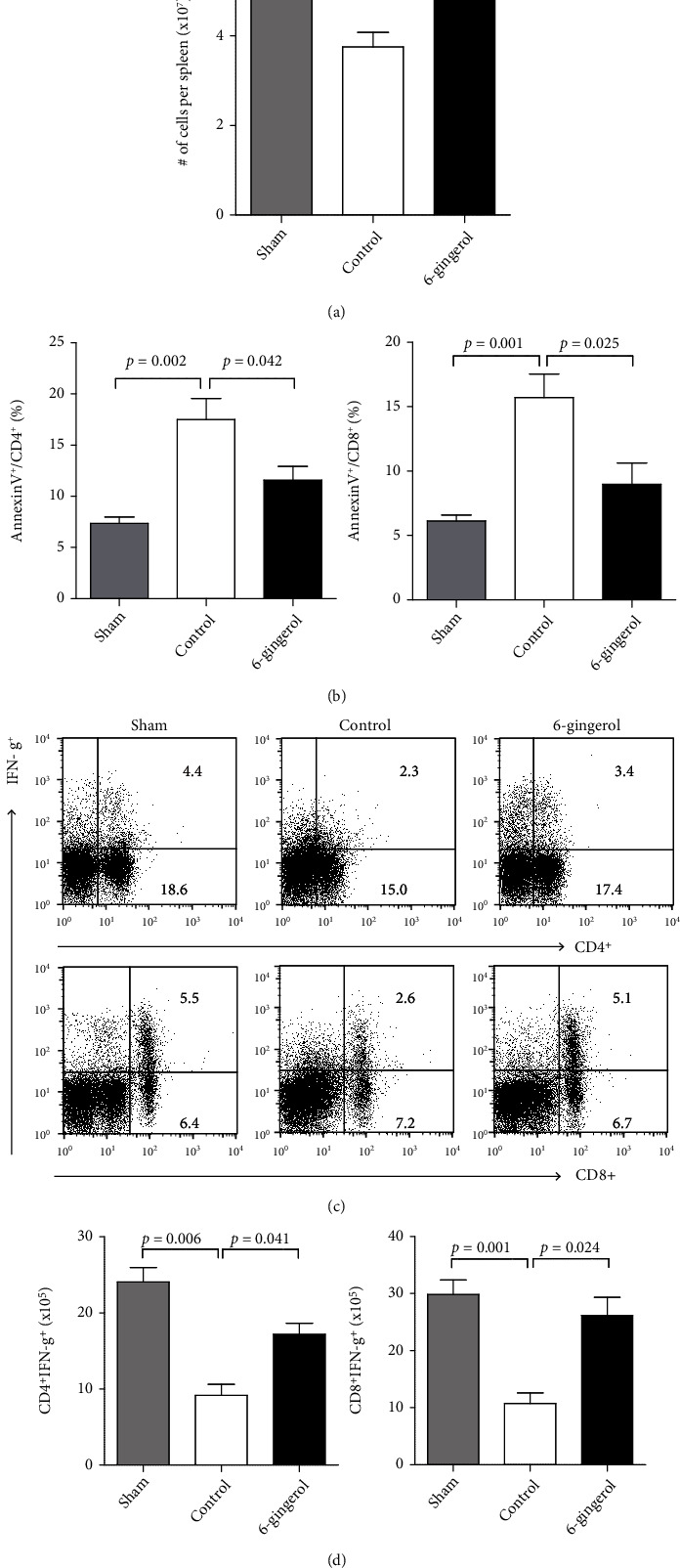
T-cells from 6-gingerol-treated mice showed significant increases in IFN-*γ* production compared to control 4 days post-CLP. Mice were subjected to CLP and fed 6-gingerol. (a) Spleen cell numbers on Day 4 after CLP. (b) Annexin V expression on CD4^+^ and CD8^+^ T-cells was analyzed by FACS. (c) Splenocytes harvested 4 days after CLP (prior to secondary injury) were evaluated by intracellular staining after ex vivo stimulation of splenocytes with PMA plus ionomycin as mentioned in the Materials and Methods. Data were analyzed on a FACSCanto™ II (BD Biosciences) with FACSDiva software. Representative flow plots showing the percentage of IFN-*γ*-producing CD4^+^ and CD8^+^ T-cells among splenocytes in sham, control, or 6-gingerol-treated mice. Panel values indicate percentage of each cell population among total cells. (d) Number of CD4^+^IFN-*γ*^+^ (left panel) and CD8^+^IFN-*γ*^+^ (right panel) cells among splenocytes in sham, control, or 6-gingerol-treated mice. Results are representative of three independent experiments. Statistically significant differences are shown on the graph.

To determine whether enhanced survival of 6-gingerol-treated mice with a secondary infection of *P. aeruginosa* was associated with the immune status of mice undergoing CLP, on Day 4, prior to secondary injury, we analyzed the spleens of 6-gingerol-treated and control mice. Lymphocyte loss is thought to be partially responsible for the profound immunosuppression seen in sepsis [2, 38, 39]. To determine whether 6-gingerol treatment was associated with decreased lymphocyte apoptosis, we evaluated spleen cell numbers on Day 4 after CLP. Splenocyte numbers in 6-gingerol-treated mice were significantly higher than those in control mice (Figure 7(a)). In addition, 6-gingerol-treated mice had a significantly lower level of annexin V expression on CD4^+^ and CD8^+^ T-cells, compared to control mice (Figure 7(b)). Next, we evaluated T-cell production of IFN-*γ* to determine the host's ability to mount an effective inflammatory response prior to secondary infection. Splenocytes harvested 4 days after CLP were stimulated with PMA (50 ng/ml) and ionomycin (500 ng/ml), and IFN-*γ*-producing CD4^+^ and CD8^+^ T-cells were analyzed by FACS. Splenocytes from 6-gingerol-treated mice demonstrated significantly enhanced percentages and numbers of IFN-*γ*-producing CD4^+^ and CD8^+^ T-cells compared to control-treated CLP mice (Figures 7(c) and 7(d)). These results indicate a better systemic immune response in the 6-gingerol-treated mice subjected to CLP surgery compared to control mice.

## 4. Discussion

Sepsis is characterized by sustained infection and uncontrolled systemic inflammatory response, which results in tissue damage and, ultimately, multisystem organ failure, the clinical hallmark of sepsis and direct cause of mortality [40]. The first host response against an invading pathogen involves the recruitment of leukocytes such as neutrophils and macrophages, to infectious foci and their activation which allows these cells to successfully localize, kill, and clear pathogens. In sepsis, bacterial signals recognized by blood neutrophils and macrophages induce the production or release of inflammatory cytokines that increase blood flow to infected tissues, enhance the permeability of local blood vessels, and recruit inflammatory cells to the site of infection [41]. In the present study, we found that 6-gingerol-treated mice exhibited reduced bacterial load in organs, had higher numbers of infiltrated peritoneal neutrophils and macrophages, and had lower levels of cell apoptosis in the CLP model when compared to control-treated mice (Figures 2(a)–2(c)).

Cytokines and chemokines play a critical role in the recruitment of leukocytes to inflamed tissues, yet they are often described as “double-edged swords.” An appropriate concentration of cytokines is necessary for the recruitment and activation of immune cells in response to foreign antigens, however, when excess cytokines are produced, they damage the host. A successful host defense response during sepsis requires a fine balance of anti- and proinflammatory cytokines [23, 42]. Several reports have demonstrated that TNF-*α*, IL-6, and IL-10 can serve as both makers and mediators of sepsis severity and that elevated cytokine levels predict mortality following CLP [43–45]. Macrophages may be excessively activated during the early phase of sepsis and produce excessive proinflammatory cytokines [46], which may be a major cause of the high mortality rate during this early stage [47]. Several studies have demonstrated that 6-gingerol inhibited proinflammatory cytokines production from LPS-stimulated macrophages [12, 13]. In our study, we found that 6-gingerol inhibited the production of TNF-*α* and IL-6 from TLR2, 4 ligand-activated macrophages as well as TNF-*α* and IL-10 from TLR2, 4 ligand-activated neutrophils (Figures 3(b) and 3(c)). Moreover, it has been documented that the plasma levels of TNF-*α* and IL-6 increased significantly in patients with sepsis and in animal models [48, 49]. In *in vivo* studies, 6-gingerol-treated mice showed a less severe inflammatory response and reduced bacterial burden in CLP-induced sepsis (Figures 2(a) and 3), indicating that the balance between efficient pathogen clearance and a harmful overactive inflammatory response was affected by 6-gingerol treatment, which may be one of the reasons for less severe organ failure and decreased mortality in these septic mice. To overcome the excessive inflammation, macrophages undergo apoptosis or polarize to an M2 phenotype to protect the host from excessive injury [50]. Given that 6-gingerol inhibited inflammatory cytokines, such as TNF-*α* and IL-6, involved in M1 macrophage polarization (Figures 3(a)–3(c)), 6-gingerol promoted M2 macrophage polarization (Figure 3(d)). As expected, 6-gingerol decreased the M1/M2 ratio (Figure 3(e)). Macrophage depletion abrogated the improved bacterial clearance in 6-gingerol-treated mice (Figure 2(d)). Therefore, our results indicate that 6-gingerol effectively prevents the development of severe sepsis after microbial infection via enhanced bacterial clearance, inhibited inflammatory cytokine production, and increased ROS production. In addition, we investigated the immunomodulatory effects of 6-gingerol on intranasal *P. aeruginosa* infection in postseptic mice. Results showed that administration of 6-gingerol improved the survival of intranasal *P. aeruginosa* infection. To investigate the role of 6-gingerol in host defense against secondary *P. aeruginosa* pneumonia, we analyzed bacteria clearance, immune cell population, apoptosis, and cytokine balance. The immunoparalysis caused by sepsis is associated with a decreased ability of the host to clear bacteria [19]. Given this, we evaluated the ability of the host to clear *P. aeruginosa* from BAL fluid and lungs following double injury. As shown in Figures 4(c) and 4(d), 6-gingerol-treated mice significantly decreased the bacterial concentration in BAL fluid and lungs 24 h after intranasal instillation. Because of this, we analyzed immune cell numbers at a much earlier time point after intranasal instillation. In BAL fluid, most infiltrated cells were neutrophils and 6-gingerol-treated mice showed significantly increased neutrophil numbers when compared to control mice. Consistent with increased neutrophil numbers, neutrophil apoptosis in 6-gingerol-treated mice was significantly reduced compared to control mice (Figure 5(d)). In addition, increased IL-17 and CXCR2 levels in 6-gingerol-treated mice compared to controls in BAL fluid suggest that 6-gingerol is also associated with neutrophil infiltration (Figures 5(e) and 5(f)). The respiratory system is continuously exposed to a variety of bacteria. To combat these intruders, one of the most important components of the initial innate immune response against bacterial infection is the vigorous recruitment of neutrophils. Neutrophils play a primary and unambiguous role in *P. aeruginosa* clearance during acute pulmonary infection, which is clearly demonstrated by the extreme susceptibility of neutropenic mice to this pathogen [51–53]. Our data indicate that 6-gingerol contributed to the effective clearance of bacteria at the site of infection due to the promotion of neutrophil recruitment and reduction in apoptosis in the hypoinflammatory state of postseptic mice.

Cytokine balance is another key mechanism related to survival. A balanced and regulated production of pro- and anti-inflammatory cytokines is essential to eradicate pathogens without tissue damage. Sepsis-induced immunosuppression is characterized by an imbalance in the expression of proinflammatory and anti-inflammatory cytokines, especially a shift towards an anti-inflammatory cytokine pattern, which predisposes the host to secondary pulmonary infections, particularly the development of nosocomial pneumonia [3, 54]. Early death in sepsis is usually a result of a hyperinflammatory response driven by proinflammatory cytokines. The early hyperinflammatory state evolves to a subsequent hypoinflammatory state with significant immunosuppression [1–3]. Researchers highlighted the importance of IL-10 as a classic anti-inflammatory cytokine [55]. During the immunosuppressive phase of sepsis, IL-10 leads to suppression of macrophage cytokine secretion and decreased activity of macrophage and neutrophil functions [55, 56]. This anti-inflammatory reaction is further characterized by low levels of circulating lymphocytes, increased lymphocyte apoptosis, and a shift from Th1 to Th2 subpopulations [57]. Previous research reported that higher plasma IL-10 concentrations contributed to higher mortality of sepsis [58, 59]. On the contrary, the importance of TNF-*α* production in response to infection has been demonstrated by multiple research groups. Lukacs et al. [60] reported that TNF-*α* mediates the recruitment of neutrophils during airway inflammation. As shown in Figure 6, 6-gingerol modulates the cytokine balance toward a proinflammatory pattern in mice with a secondary infection of *P. aeruginosa*. These data suggest that 6-gingerol treatment improves host defense by removing bacteria. Several studies have demonstrated that 6-gingerol inhibited the production of proinflammatory cytokines from LPS-stimulated macrophages [12, 13]. In our model, 6-gingerol treatment reduced hyperinflammation in CLP mice, thereby preventing them from moving towards the immunosuppressive phase.

Multiple research studies using animal models and patients dying from sepsis have demonstrated a profound loss of immune effector cells by apoptosis [2, 38, 61]. In order to evaluate the immune status of mice undergoing CLP, on Day 4, prior to secondary injury, we analyzed the spleens of 6-gingerol-treated and control mice. As shown in Figures 7(a) and 7(b), splenocyte numbers in 6-gingerol-treated mice were significantly higher, while the levels of T-cell apoptosis were lower than in control mice. In addition, splenocytes from 6-gingerol-treated mice exhibited enhanced T-cell production of IFN-*γ* compared to control mice (Figure 7(c)). IFN-*γ* treatment has been shown to improve the clinical course of sepsis in humans [62]. Hotchkiss and Karl [2], in adoptive transfer experiments, showed that increased animal survival was due to IFN-*γ* upregulation. Therefore, the reduction in T-cell apoptosis and improvement in IFN-*γ* production ability in 6-gingerol-treated mice indicate a better systemic immune response than those in control mice.

## 5. Conclusion

In conclusion, our results indicate that 6-gingerol effectively prevents the development of severe sepsis after microbial infection via enhanced bacterial clearance and inhibited inflammatory cytokine production. In addition, we found that 6-gingerol improves survival rates from infection in a murine model of sepsis-induced immune dysfunction. Survival improvement was associated with enhanced neutrophil numbers and function early on the infection site, a shift from a Th2 to a Th1 cytokine balance in the hypoinflammation phase, and decreased lymphocyte apoptosis.

## Data Availability

The data used to support the findings of this study are included within the article.
